# *Echinococcus granulosus* antigen B ameliorates myocardial infarction through promoting M2 macrophage polarization

**DOI:** 10.3389/fcimb.2025.1662758

**Published:** 2025-11-03

**Authors:** Weixiao Zhang, Bingxin Liu, Sai Wang, Xinlong Xu, Qiwang Jin, Chen Yang, Hui Wang, Erhe Gao, Bin Zhan, Shili Wu, Rui Wang, Rui Zhou, Xiaodi Yang

**Affiliations:** ^1^ First Affiliated Hospital of Bengbu Medical University, Bengbu, China; ^2^ Anhui Key Laboratory of Infection and Immunity of Bengbu Medical University, Bengbu, China; ^3^ Department of Environmental Genomics, Jiangsu Key Laboratory of Cancer Biomarkers, Prevention and Treatment, Collaborative Innovation Center for Cancer Personalized Medicine, Nanjing Medical University, Nanjing, China; ^4^ Lewis Katz School of Medicine, Temple University, Philadelphia, PA, United States; ^5^ National School of Tropical Medicine, Baylor College of Medicine, Houston, TX, United States; ^6^ Scientific Research Center, Bengbu Medical University, Anhui, Bengbu, China; ^7^ Basic Medical College of Bengbu Medical University, Bengbu, China

**Keywords:** myocardial infarction, *Echinococcus granulosus*, antigen B (EgAgB), macrophage, NOD-like receptor family pyrin domain-containing 3 (NLRP3)

## Abstract

**Background:**

Myocardial infarction (MI) is a severe cardiovascular condition arising from a sudden reduction or complete cessation of blood supply via the coronary arteries, resulting in localized necrosis of the corresponding myocardial tissue due to persistent ischemia and hypoxia. It is a life-threatening cardiovascular disease with high death rate and poor prognosis due to myocardial necrosis. *Echinococcus granulosus* hydatid cyst-secreted antigen B (*Eg*AgB) has been shown to play a critical role in modulating host immune responses. This study aims to investigate whether the *Eg*AgB subunit 8 (*Eg*AgB8/2) is able to mitigate inflammation associated with myocardial infarction and therefore reduce the MI caused mortality in a mouse model,. The MI model was established by ligating the left anterior descending (LAD) coronary artery in male C57BL/6J mice, followed by intraperitoneal administration of recombinant *Eg*AgB subunit 2 (r*Eg*AgB8/2) to observe its therapeutic effect on MI and related immunological mechanism. To evaluate the role of r*Eg*AgB8/2 in macrophage polarization post-MI, mRNA levels of M1 macrophage marker iNOS and M2 marker *Arg-1* were determined in infarcted regions using RT-qPCR. *In vitro*, RAW264.7 macrophages were co-incubated with rEgAgB8/2 and observe whether r*Eg*AgB8/2 is able to promote M2 macrophage polarization under inflammation condition.

**Results:**

Our study in a MI mouse model demonstrated that treatment with r*Eg*AgB8/2 significantly improved cardiac function and survival rates from 66.7% to 94.4% within 28 days post MI surgery compared to MI group without treatment. The levels of pro-inflammatory cytokines TNF-α and IL-1β were significantly reduced in infarcted region in heart tissue and serum, while regulatory cytokines IL-10 and TGF-β were increased following r*Eg*AgB8/2 treatment associated with reduced expression of M1 macrophage marker iNOS and increased expression of the M2 macrophage marker Arg-1. The treatment of r*Eg*AgB8/2 downregulated NLRP3, caspase-1, and IL-1β protein levels in infarcted tissues.

*In vitro* study with macrophage cell line RAW264.7 further demonstrated that co-incubation of r*Eg*AgB8/2 with LPS-induced RAW264.7 cells resulted in a decrease in the proportion of CD86^+^ macrophages (M1) and an increase in the proportion of CD206^+^ macrophages (M2) associated with reduced inflammatory cytokines (TNF-α and IL-1β) and increased regulatory cytokines (IL-10 and TGF-β), which was consistent with the results obtained from the *in vivo* experiments in a MI mouse model.

**Conclusions:**

These findings reveal that r*Eg*AgB8/2 ameliorates MI in mice by promoting macrophage polarization from M1 to M2 phenotypes through inhibition of the NLRP3/caspase-1/IL-1β signaling pathway, indicating its potential as a therapeutic agent for MI and other inflammation-related diseases.

## 1 Introduction

Myocardial infarction (MI), commonly known as a heart attack, is a serious medical emergency in which the supply of blood to the heart is suddenly reduced or blocked causing the tissue in the heart muscle to die (Negrini et al., 2020; Salari et al., 2023). It is the most common cause of heart failure with high mortality (Roger, 2013). The incidence of MI has been increasing year by year worldwide, imposing a growing burden on healthcare services (Roger, 2013; The W. C. O. T. R. O. C. H. A. D. I. C, 2023; Zhang et al., 2025).

An increasing number of studies have demonstrated that the process of MI is accompanied by an inflammatory response (Zhang et al., 2022). Inflammation after MI occurs due to complex mechanisms (Nahrendorf et al., 2010; Arslan et al., 2011; Yan et al., 2013; Frangogiannis, 2014; Frangogiannis, 2015; Prabhu and Frangogiannis, 2016; Shiraishi et al., 2016; Hulsmans et al., 2018; Shirakawa et al., 2018; Mouton et al., 2019; Daseke MJ et al., 2021; Kologrivova et al., 2021; Zhang et al., 2022; Wu et al., 2023). Dead myocyte released damage-associated molecules patterns (DAMPs) that serve as warning signals (Arslan et al., 2011) to activate innate immune system. The neutrophils are first recruited to the infarct site as the sharpest sentinels to remove damaged cell debris and initiate repair processes (Daseke MJ et al., 2021). Afterwards, monocytes are recruited and differentiated into different macrophage subpopulations, with M1-type macrophages promoting inflammation especially during the acute phase (Yan et al., 2013; Frangogiannis, 2014; Prabhu and Frangogiannis, 2016), and then M2-type macrophages contributing to tissue repair and inflammation abatement during the recovery phase (Nahrendorf et al., 2010; Shiraishi et al., 2016; Hulsmans et al., 2018; Shirakawa et al., 2018; Mouton et al., 2019; Wu et al., 2023). Persistent or excessive inflammation can result in augmented fibrosis, thereby impairing cardiac function and elevating the risk of heart failure (Frangogiannis, 2015; Kologrivova et al., 2021). Therefore, reducing the inflammatory responses during the acute phase and regulating the balance between M1 and M2 macrophages is crucial for reducing mortality of MI and improving MI outcomes in the treatment of MI.

In recent years, multiple studies have found that helminth infections modulate host immune responses with biased Th2 and regulatory responses that reduce host immune attack on the parasite as a survival strategy (van Riet et al., 2007; McSorley et al., 2013). This immunomodulatory ability during infection usually takes place through secreting some biofunctional molecules that inhibit host Th1-dominated inflammatory responses and induce regulatory Th2 or macrophage M2-related tissue repair and recovery (Xie et al., 2021; Wu et al., 2023). These immunomodulatory functions of helminth infection and helminth-derived products have been experimentally or clinically used to treat some auto-immune or inflammatory diseases (Yang et al., 2014; Elliott and Weinstock, 2017; Li et al., 2017; Gao et al., 2020; Li et al., 2021; Li et al., 2022). *Echinococcus granulosus* is a tapeworm in dog. Human become infected by ingesting eggs from the feces of infected dogs to cause cystic echinococcosis (CE), a globally widespread zoonotic disease (Wang et al., 2021). *E. granulosus* antigen B (*Eg*AgB) is the major protein secreted in the cystic fluid by metacestode larvae with multiple functions involved in key host-parasite interactions during infection (Wang et al., 2023). *Eg*AgB is a polymeric lipoprotein of 160 kDa which consists of different isoforms of 8-kDa proteins encoded by a polymorphic multigene family comprising five subfamilies including *Eg*AgB8/1, *Eg*AgB8/2, *Eg*AgB8/3, *Eg*AgB8/4 and *Eg*AgB8/5 (Mamuti et al., 2007; Obal et al., 2012). Due to its high antigenicity, *Eg*AgB has been widely used in the immunodiagnosis of cystic *echinococcosis* (CE) (Khalilpour et al., 2014; Biosa et al., 2022), especially recombinant *Eg*AgB8/2 protein exhibited higher diagnostic value in comparison with other recombinant *Eg*AgB subunits or native *Eg*AgB for the serodiagnosis of human CE (Savardashtaki et al., 2019; Poggio et al., 2022). Except for their immunodominant property during infection used as an immunodiagnostic antigen, *Eg*AgB and its subunits also interact with host immune cells and possess immunomodulatory properties by binding to monocytes and macrophages and directly inhibiting the production of pro-inflammatory cytokines to reduce host’s inflammatory response (Al-Aghbar et al., 2008; Boutennoune et al., 2012; Silva-Álvarez et al., 2016; Folle et al., 2024). Based on these findings, the *Eg*AgB was experimentally used to treat inflammatory bowel disease, resulting in a significant reduction in bowel inflammation through regulating M1/2 polarization (Bao et al., 2022). Additionally, our previous study demonstrated the therapeutic effect of *Echinococcus granulosus* cyst fluid on bacterial sepsis in a mouse model (Wang et al., 2023).

Our previous studies demonstrated that excretory/secretory proteins from *Trichinella* sp*iralis* adult worms could improve MI in mice by modulating macrophage differentiation into M2 type (Wu et al., 2023). However, the therapeutic effects of *Eg*AgB on cardiovascular disease, especially MI, have not been studied. In this study we assessed the therapeutic efficacy of recombinant *Eg*AgB8/2 in MI caused heat damage and mortality, elucidated the underlying immunological mechanisms, with a goal to develop r a novel therapeutic strategy to treat myocardial infarction and other inflammation-related diseases.

## 2 Methods

### 2.1 Animals

The specific pathogen-free male C57BL/6J mice (6–8 weeks old, weighing 18–22 g) were purchased from the Experimental Animal Center of Bengbu Medical University. All mice were housed in a temperature- and humidity-controlled room with a 12-hour light/dark cycle, free access to standard rodent chow and water. The temperature of the room was maintained ranged from 22-24 °C, with humidity kept at 40-60%.Mice were randomly assigned to different groups using a random number table method, and all analyses were performed in a blinded manner, where the investigators were unaware of the groups.

All procedures were conducted in accordance with the guidelines established by the Ethics Committee of Bengbu Medical University (approval number: BBMC-2024-617). To be operated solely by trained personnel to avoid causing distress to mice through improper handling. The specific operational procedures are as follows:

Anesthesia: Mice were induced under anesthesia using 2% isoflurane (RWD, Shenzhen). During surgery, the R500 Series Universal Small Animal Anaesthesia Machine (RWD, Shenzhen) was maintained via an anesthesia machine, with oxygen flow set at 1 L/min to ensure stable anesthetic depth.

Euthanasia: At the experimental endpoint, mice were subjected to progressive exposure to carbon dioxide (flow rate: 20% of chamber volume per minute) until respiratory arrest occurred. Cervical dislocation was then employed as a secondary confirmation method.

### 2.2 Preparation of recombinant *E. granulosus* antigen B subunit 2 protein (r*Eg*AgB8/2)

ZOON-BIO BIOTECHNOLOGY synthesized the gene sequence encoding *Eg*AgB subunit 2 (GenBank: ACZ51457) and cloned into *E. coli* expression vector pPET-28a(+) through homologous recombination with 8His-tag expressed at C-terminus. The correct sequence and reading frame of recombinant *Eg*AgB8/2/pPET-28a(+) was confirmed by double-strand DNA sequencing. The recombinant plasmid was transformed into *E. coli* BL21(DE3) and r*Eg*AgB8/2 was expressed under induction of 1 mM IPTG at 30 °C for 4 hours. The expressed r*Eg*Ag8/2 was soluble and purified through nickel-affinity chromatography. The contaminated endotoxin in the purified protein was removed using the ToxOut Endotoxin Removal Kit (BioVision, USA), and residual endotoxin levels were measured with the ToxinSensor Chromogenic LAL Endotoxin Assay Kit (GenScript Biotech, Nanjing, China). The purified r*Eg*AgB8/2 was stored in PBS buffer without imidazole, and its concentration was determined using a BCA Protein Assay Kit (Biosharp, Hefei). SDS-PAGE was used to confirm the expression and purity of the target protein. The purified r*Eg*AgB8/2 was subsequently stored at -80 °C for future use.

### 2.3 MI mouse model

Coronary artery ligation is a well-established method to create a MI model in mice. In the sham group, the same surgical procedure was performed, except the LAD was not ligated. In this study, we used a novel and rapid surgical approach that does not require mechanical ventilation (Gao et al., 2010). Mice were anesthetized with 2% isoflurane via inhalation, and their respiration was closely monitored throughout the procedure. A small skin incision (approximately 5 mm) was made between the 4th and 5th ribs on the left side of the mouse chest, followed by gentle separation of the intercostal muscles to access the thoracic cavity. The left anterior descending (LAD) coronary artery was identified and ligated 3 mm distal to its origin using a 6–0 silk suture. Successful ligation was confirmed by the anterior wall of the left ventricle turning pale, indicating interruption of blood flow. If a vein is mistakenly ligated, typical myocardial blanching does not occur; instead, local congestion (dark purple area) may develop due to obstructed venous return. This characteristic allows direct identification and exclusion of mice with erroneous vein ligation. In addition to cases of incorrect vein ligation, four categories of mice must be excluded from myocardial infarction modelling: those exhibiting severe postoperative complications such as hemorrhage, pneumothorax, arrhythmia, or respiratory distress; those displaying severe infections at the thoracic incision site, including redness, swelling, or suppuration; and those experiencing weight loss exceeding 20% or significant loss of locomotor function within one week post-surgery. One week postoperatively, there was no significant decrease in left ventricular ejection fraction (LVEF) (difference<10% compared to the sham-operated group). Mice that showed no typical signs of myocardial infarction, as verified by TTC staining or HE staining, were also excluded. All animals were monitored for their survival or recovery.

### 2.4 Treatment of MI mice with r*Eg*AgB8/2

C57BL/6 mice were randomly divided into four groups with 36 mice for each group: (i) Sham operation control treated with PBS (Sham+PBS); (ii) Sham control treated r*Eg*AgB8/2 (Sham+r*Eg*AgB8/2); (iii) MI group treated with PBS (MI+PBS); (iv) MI group treated with r*Eg*AgB8/2 (MI+r*Eg*AgB8/2). Mice were administered intraperitoneally with 5 µg of r*Eg*AgB8/2 in total volume of 100 µl for treatment or the same volume of PBS 30 minutes after surgery. The same amount of r*Eg*AgB8/2 or PBS was given 2, 4, and 6 days post operation. Mice were monitored daily for weight, behavior, and general health status. Cardiac function was evaluated using echocardiography at days 7 to assess myocardial function and progression of MI. Total 18 mice were sacrificed on Day 7 post-surgery, the blood and sera were collected for cytokine measurement and heart tissues collected for biochemical and histological examination including mRNA measurement of some immunological molecules. The rest 18 mice from each group were kept for long-term survival observation till Day 28. The survival rate was calculated using the Kaplan-Meier method. Mice meet the criteria for humane endpoints included a body weight loss for more than 20% or signs of severe distress will be euthanized in accordance with ethical guidelines.

### 2.5 Triphenyl tetrazolium chloride staining of infarct hearts

To evaluate infarct size after MI, the hearts collected from 6 sacrificed mice per group on Day 7 were rinsed in saline and quickly frozen at −20 °C for 10–15 minutes to allow for easier slicing without over-freezing. Each heart was then cut into 5 slices, each 1–2 mm thick, from the ligation point to the apex. The heart slices were incubated in 1% 2,3,5-triphenyltetrazolium chloride (TTC) staining solution at 37 °C for 10–20 minutes in the dark. The white area indicates the infarcted tissue, while the red area indicates viable, non-infarcted tissue. The stained slices were photographed, and infarct size was measured using ImageJ software.

### 2.6 Hematoxylin and eosin staining and Masson’s trichrome staining of heart tissue

The hearts from 6 sacrificed mice per group were perfusion-fixed using 10 mL of ice-cold PBS at a flow rate of 5 mL/min and then stored in 4% paraformaldehyde at 4 °C for 24 hours. After fixation, hearts were embedded in paraffin blocks and sliced into 5-μm-thick sections. The sections were stained with H&E (Servicebio, China) or Masson’s Trichrome (Servicebio, China) as described (Li et al., 2021). Subsequently, histopathological changes were observed under microscope (Nikon, Japan) and the pathological area was analyzed using ImageJ software. Regions of interest were standardized across samples, analyzing 10 random fields per section, with threshold settings adjusted to highlight specific tissue features. Data were statistically analyzed to quantify histopathological differences between groups.

### 2.7 Echocardiography

Echocardiography (ECHO) was performed for all mice on Day 7 post surgery using the Vevo 2100 imaging system paired with a probe (VisualSonics, Canada, MS-400 probe). Before echocardiography was performed on mice, the chest area was shaved, and ultrasound coupling gel was applied to the skin to improve contact between the probe and the skin. Mice were positioned supine on a thermostatic platform, and electrodes were attached to the limbs for continuous monitoring of heart rate, respiratory rate, and oxygen saturation. Superficial anesthesia was maintained with 1% isoflurane in oxygen, and heart rate was monitored throughout the procedure to ensure it remained within a controlled range of 400–500 bpm across all animals. Long-axis B-mode imaging was used to acquire left ventricular images, and M-mode tracings of the left ventricular wall were recorded for 4–6 continuous and stable cardiac cycles. The best 3 cycles, defined by their stability and consistency, were selected for analysis. The left ventricular trace method was applied to obtain stroke volume (SV), left ventricular ejection fraction (LVEF), and left ventricular fractional shortening (LVFS). All values were averaged over the selected cardiac cycles. Ultrasound imaging parameters, such as probe frequency and frame rate, were standardized for all mice to ensure consistency in data collection. Data analysis was performed blindly by a separate investigator who was unaware of the experimental groups. Heart function indices were calculated using the following equations:


LVEF (%) = ((LVEDV - LVESV)/LVEDV) * 100%

LVFS (%) = ((LVIDd - LVIDs)/LVIDd) * 100%

Measurements were taken using automated tracing algorithms to minimize human error, and heart rate was maintained at a consistent level to avoid confounding effects on the measurements.

### 2.8 Enzyme−linked immunosorbent assay

Serum samples were used directly, while heart tissue extracts were prepared by homogenizing in ice-cold PBS (1 mL per 100 mg tissue) and centrifuging at 10,000 xg. Cytokine levels were measured in the sera and heart extracts using individual ELISA kits: Mouse TNF-α, IL-1β, and IL-10 ELISA Kits (Dakewe Biotech, China); Mouse IL-18, iNOS, and *Arg-1* ELISA Kits (RUIXIN Biotech, China); and Mouse TGF-β ELISA Kit (ABclonal, USA). All samples and standards were incubated for two hours at room temperature, plates washed three times with PBS after each incubation. Results were read using a microplate reader at a wavelength of 450 nm. Data were analyzed using the standard curve method to calculate cytokine concentrations in each sample, with statistical analysis performed using one-way analysis of variance (ANOVA).

### 2.9 RNA extraction and real-time quantitative PCR

Total RNA was extracted from 100 mg of cardiac infarction tissue using TRIzol reagent (TransGen Biotech, China), following tissue disruption with a rotor-stator homogenizer at 6,000 rpm for 30 seconds. RNA purity was assessed by spectrophotometry, with A260/A280 ratio between 1.8 and 2.1. For reverse transcription, 2 μg of total RNA was used in a 20 μL reaction using reverse transcription kits (TransGen Biotech, China). RT-qPCR was performed using PerfectStart^®^Green qPCR SuperMix (TransGen Biotech, China) on a Roche LightCycler^®^ 96 system. The specific annealing temperatures and extension times for each primer set (listed in **Table 1**) were optimized based on primer efficiency tests. GAPDH was used as the internal control, validated under experimental conditions to ensure stable expression. The relative mRNA expression of target genes was calculated using the 2−△△Ct method, and average Ct values were used for calculations.

**Table 1:** The related primers of target genes used in qPCR.

ID	Primer sequences (5’→3’)
GAPDH-F	GGTTGTCTCCTGCGACTTCA
GAPDH-R	TGGTCCAGGGTTTCTTACTCC
TNF-α-F	CGAGTGACAAGCCTGTAGCC
TNF-α-R	ACAAGGTACAACCCATCGGC
IL-10-F	GGTTGCCAAGCCTTATCGGA
IL-10-R	AATCGATGACAGCGCCTCAG
TGF-β-F	CTGGATACCAACTACTGCTTCAG
TGF-β-R	TTGGTTGTAGAGGGCAAGGACCT
IL-1β-F	TGCCACCTTTTGACAGTGATG
IL-1β-R	GCTCTTGTTGATGTGCTGCT
NLRP3-F	ATTACCCGCCCGAGAAAGG
NLRP3-R	CATGAGTGTGGCTAGATCCAAG
Caspase-1-F	TGCTACGCTCCGAATCTA
Caspase-1-R	GTTCCACATCTGACTTAGGT
iNOS-F	CAAGCACCTTGGAAGAGGAG
iNOS-R	AAGGCCAAACACAGCATACC
Arg-1-F	TGTCCCTAATGACAGCTCCTT
Arg-1-R	GCATCCACCCAAATGACACAT
α-SMA-F	AGCGGGCATCCACGAAAC
α-SMA-R	TTGATCTTCATGGTGCTGGGT
VEGF-F	CCCACGTCAGAGAGCAACAT
VEGF-R	TGCGCTTTCGTTTTTGACCC

### 2.10 Western blot

Total protein was extracted from heart tissues using RIPA lysis buffer (100 μL per 10 mg of tissue) containing 0.1% PMSF. Samples were lysed with agitation for 30 minutes at 4°C. Protein concentrations were determined by a BCA Protein Quantitation Kit, using a standard curve prepared from known concentrations of bovine serum albumin. Equal amounts of protein from each mouse were loaded, separated by 12.5% SDS-PAGE gels, and then transferred onto 0.45 µm polyvinylidene fluoride (PVDF) membranes. The membranes were blocked with 5% skim milk at room temperature for 3h and then incubated with rabbit anti-NLRP3 antibody (1:1,000) (Wanleibio, Shenyang, China), rabbit anti-Pro-Casepase-1 antibody (1:1,000) (Wanleibio, Shenyang, China), rabbit anti-Cleaved-Casepase-1 antibody (1:1,000) (Wanleibio, Shenyang, China), rabbit anti-IL-1β antibody (1:1,000) (Wanleibio, Shenyang, China) overnight at 4 °C followed by incubation with goat anti-rabbit IgG-HRP secondary antibody (1:7,000) (Abcam, Cambridge, UK) at room temperature for 1h. The same amount of tissue lysate was reacted with rabbit anti-glyceraldehyde-3-phosphate dehydrogenase (GAPDH) antibody (1:1,000) (Wanleibio, Shenyang, China) as baseline control. The specific bands were visualized by chemiluminescent substrate and semi-quantitated by Image Lab System. The results are expressed as ratios of NLRP3, Pro-Casepase-1, Cleaved-Caspase-1 and IL-1β to GAPDH control.

### 2.11 Cell culture and treatment

RAW264.7 cells were purchased from Procell (Wuhan, China) and cultured in Dulbecco’s modified Eagle medium (DMEM; HyClone, USA) containing 10% fetal bovine serum (FBS; Gibco, USA) and 1% Penicillin-Streptomycin solution (P/S; Biosharp, China). Cells were maintained at 37 °C in a 5% CO2 humidified incubator.

### 2.12 Effect of r*Eg*AgB8/2 on macrophage polarization *in vitro*

Harvested RAW264.7 cells were divided into 4 groups with 1×10^6^ cells in each group: (i) RAW264.7 cells incubated with PBS as the control group (PBS+Mφ); (ii) RAW264.7 cells incubated with r*Eg*AgB8/2 (1 μg/mL) (r*Eg*AgB8/2+Mφ); (iii) RAW264.7 cells incubated with LPS (100 ng/mL) (LPS+Mφ); (iv) RAW264.7 cells incubated with LPS (100 ng/mL) in the presence of r*Eg*AgB8/2 (1 μg/mL) (r*Eg*AgB8/2+LPS+Mφ). After 24 hours incubation, the cells and culture supernatants were collected for subsequent experiments.

### 2.13 Flow cytometry

Cultured RAW264.7 cells were adjusted to a density of 1×10^6^ cells/mL for staining procedures. The following three markers were primarily selected. F4/80: A pan-macrophage surface marker widely used for specific identification of macrophages in various mouse tissues. CD86: A key co-stimulatory molecule highly expressed on M1- polarized macrophages (pro-inflammatory phenotype). CD206 (mannose receptor): a classic marker for M2-polarized macrophages (anti-inflammatory/regulatory phenotype). Cells were first treated with fixable viability dye efluor 510 (BioLegend, USA) at a concentration of 0.5 µg/mL for 10 minutes in the dark to differentiate dead from live cells, followed by blocking with Fc receptor blocker (0.5 µg/mL) for 15 minutes to minimize non-specific antibody binding. Surface markers were stained with FITC-anti-F4/80 and APC-anti-CD86 antibodies (all at 1:200 dilution, BioLegend, USA) for 25 minutes. To accurately quantify total CD206 (surface + intracellular) as an indicator of M2 macrophage polarization, permeabilization is required to allow the antibody to enter the cell and bind intracellular CD206. Cells were fixed and permeabilized using the Thermo Fixation/Permeabilization Kit (Thermo Fisher Scientific, USA) for 20 minutes at room temperature. Subsequently, cells were stained with PE-anti-CD206 (1:200 dilution, BioLegend, USA) for 30 minutes in the dark. Flow cytometry data were acquired on a DxP Athena™ flow cytometer (Cytek Biosciences Inc., CA, USA), collecting a minimum of 10,000 viable cells per sample. Compensation was adjusted for FITC, BV605, APC, and PE to correct for spectral overlap. Data analysis was performed using FlowJo V10 software, focusing on the quantification of surface expression levels relative to controls.

### 2.14 Single-cell RNA sequencing

Heart tissue samples were processed for single-cell RNA sequencing. Approximately 16,000 cells were loaded onto a single channel of the 10X Genomics Chromium controller, aiming to recover 10,000 cells. Single-cell gel bead-in-emulsions (GEMs) were generated using Chromium v2 and v3 single-cell reagent kits, followed by cDNA synthesis and 11 cycles of amplification using a C1000 Touch Thermal Cycler. Quality control and quantification were performed using the Agilent 2100 Bioanalyzer. A total of 50 ng of amplified cDNA was used for constructing 3’ expression libraries, which were then pooled and sequenced on an Illumina HiSeq 4000 with 150 bp paired-end reads. The resulting basecall files were converted to FASTQ format and aligned to the murine genome (mm10) using the CellRanger v3.0.2 pipeline. Initial cell processing was conducted using the Seurat R package (v3.0.2), with data normalization and integration via canonical correlation analysis and mutual nearest neighbors (MNN). UMAP dimensionality reduction was performed with the top 30 calculated dimensions and a resolution of 0.6. Cell type identification was achieved using the SingleR R package, correlating single-cell expression profiles with the Immgen database. Differential gene expression analysis was conducted using the Wilcoxon rank-sum test, with additional analysis across clusters using the Seurat FindAllMarkers function (log-fold change > 0.25, minimum group percentage 10%, pseudocount 0.1). Bonferroni correction was applied for multiple hypothesis testing. Gene set enrichment analysis was performed using the escape R package, and differential enrichment was determined using the getSignificance function based on the limma linear fit model.

### 2.15 Statistical analysis

All treatments and analyses were performed in a blinded manner, with researchers unaware of group assignments during both treatment administration and outcome assessment. Statistics were analyzed using GraphPad Prism version 7 software (GraphPad Software, Inc., USA). All samples were randomly grouped to minimize bias between groups, and the data were tested for normality and homogeneity of variance using Shapiro-Wilk and Levene’s tests, respectively. Results are presented as means ± SEM. Multiple group comparisons were analyzed using ANOVA, with *post-hoc* adjustments for multiple comparisons made using Tukey’s test. The primary comparison will be between the Sham+PBS group and the MI+PBS group to demonstrate differences following modelling. The MI+r*Eg*AgB8/2 group will be compared with the MI+PBS group to demonstrate therapeutic efficacy. Kaplan-Meier survival analysis was conducted, and survival rates between groups were compared using the log-rank test. Statistical significance was set at *P*< 0.05. All experiments were conducted with at least three biological replicates.

## 3 Results

### 3.1 Expression and purification of r*Eg*AgB8/2

**Figure 1:**
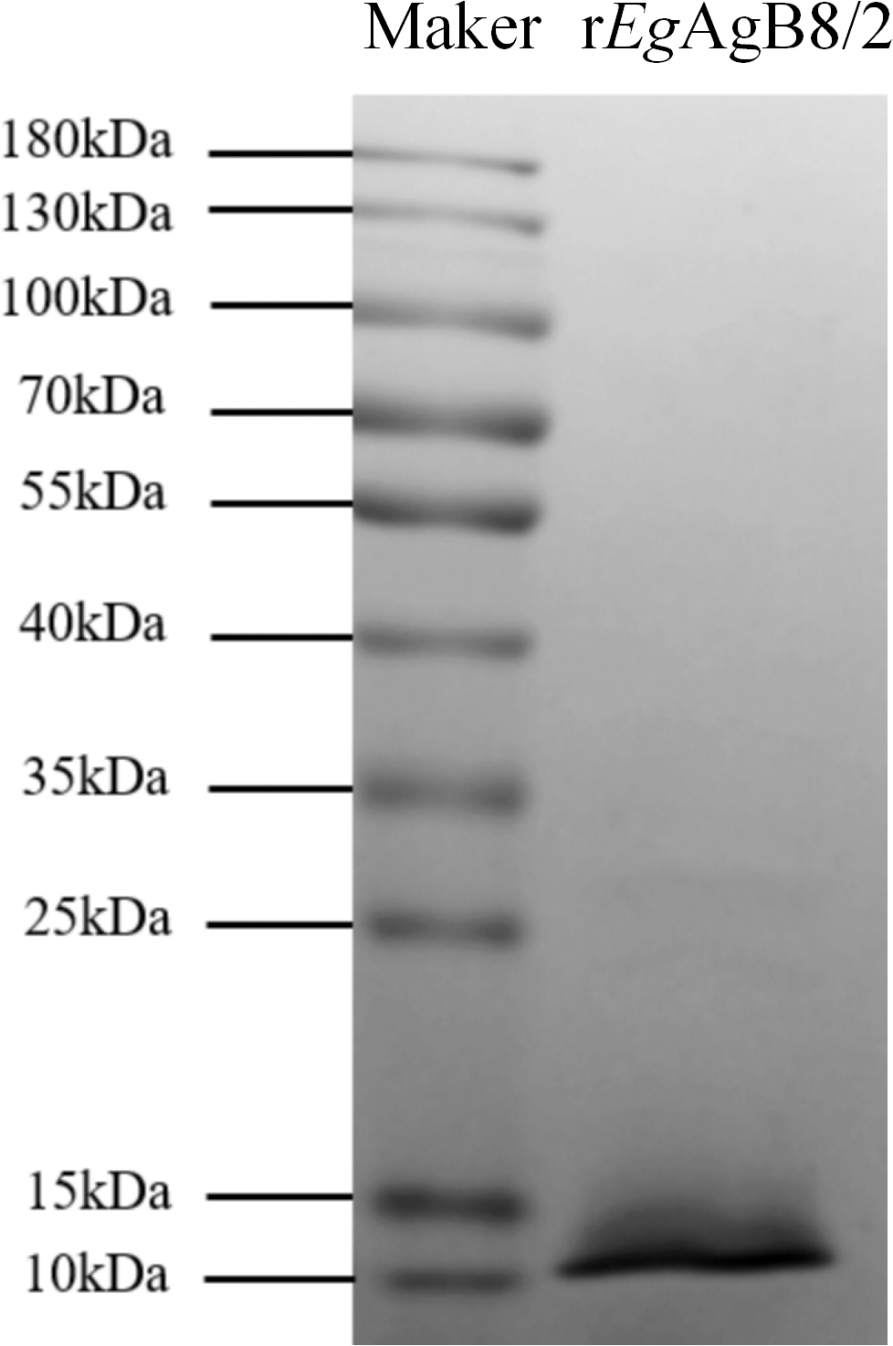
SDS-PAGE of purified r*Eg*AgB8/2. Total 3μg of purified r*Eg*AgB8/2 was separated by 12% polyacrylamide gel electrophoresis.

r*Eg*AgB8/2 with 8His-tag at C-terminus was successfully expressed as a soluble recombinant protein in *E. coli* BL21(DE3) and purified through nickel-affinity chromatography. The contaminated endotoxin was removed by running through endotoxin removal resin and the final endotoxin level of r*Eg*AgB8/2 was reduced to less than 0.1 EU/mg. SDS-PAGE showed that the purified r*Eg*AgB8/2 was approximately 10 kDa in size (including 8His-tag), which was consistent with the deduced theoretical molecular weight (9.4 kDa) (**Figure 1**).

### 3.2 Treatment with r*Eg*AgB8/2 alleviated MI in mice

**Figure 2:**
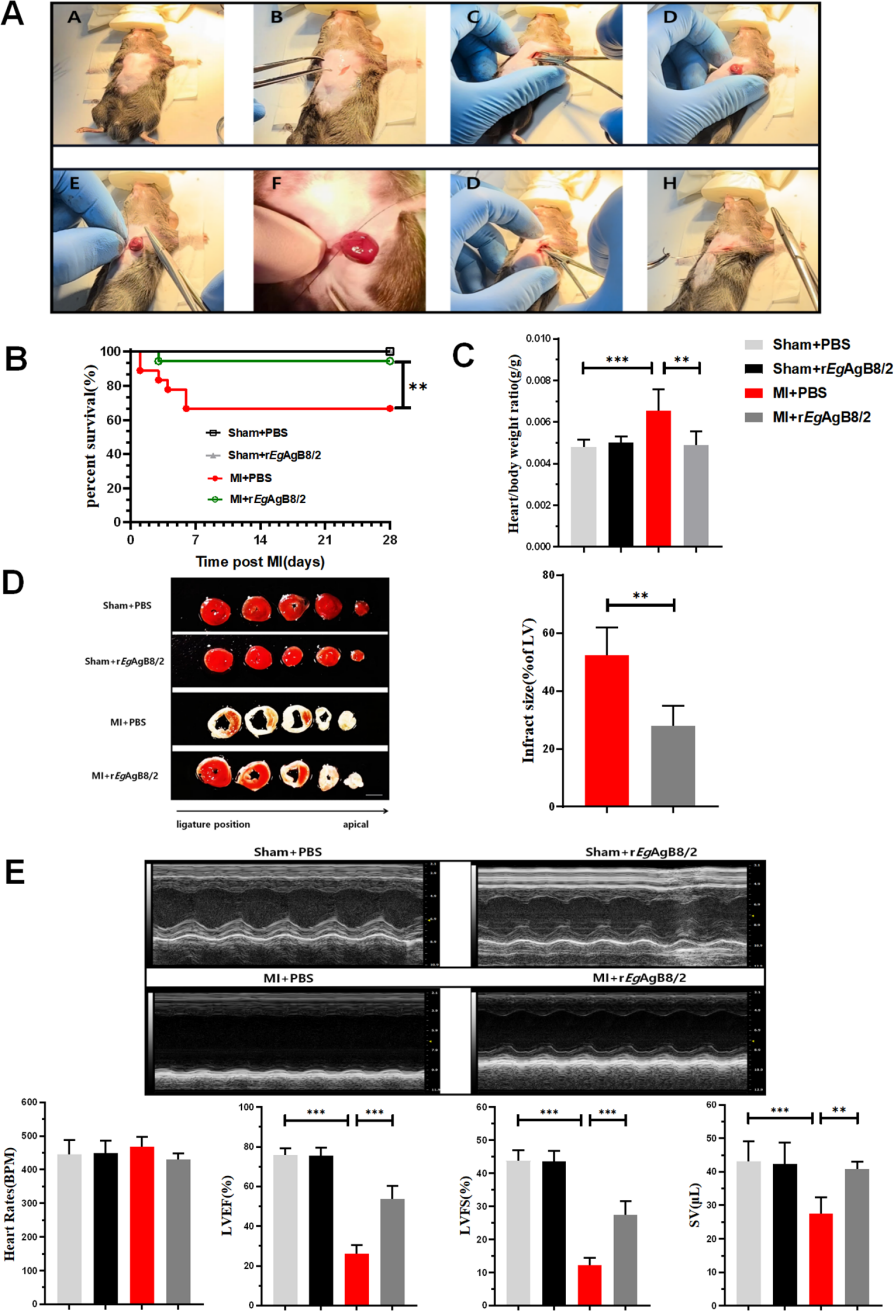
r*Eg*AgB8/2 alleviated MI in mice. **(A)** The mouse model of MI was induced by ligating the LAD coronary artery as described in Methods and Materials. **(B)** r*Eg*AgB8/2 significantly increased the survival rate from 66.67% in untreated (PBS) MI group 66.667% (12/18) in the control group with PBS only to 94.4% in treated group (17/18) over a 28-day period. Sham surgery did not cause any death. **(C)** r*Eg*AgB8/2 treatment reduced the heart-to-body weight ratio in mice post-MI surgery to the similar level of mice with sham surgery, indicating decreased cardiac edema and hypertrophy (n = 6). **(D)** Treatment with r*Eg*AgB8/2 significantly reduced the infarct size in MI mice, highlighting its cardioprotective effects (n=5). **(E)** A representative of echocardiography showing r*Eg*AgB8/2 treatment improved the motion of the anterior and posterior walls of the left ventricle, significantly enhancing LVEF, LVFS, and SV indices in MI mice (n=5). Note: Data are presented as mean ± SEM. Statistical significance is indicated as *P< 0.05, **P< 0.01, ***P< 0.001.

MI was induced in mice by ligating the LAD artery as described (Gao et al., 2010) (**Figure 2A**), followed by a 7-day consecutive treatment with r*Eg*AgB8/2 via intraperitoneal injection, as previously described (Boutennoune et al., 2012). Kaplan-Meier survival analysis indicated that the post-MI survival rate of mice treated with r*Eg*AgB8/2 (MI+r*Eg*AgB8/2) remained 94.4% (17/18) at Day 28, which is significantly higher than MI group without treatment (66.667%,12/18) (MI+PBS) within the same period (**Figure 2B**). The ratio of heart weight to body weight, as a marker for heart edema and inflammation, was also significantly reduced in r*Eg*AgB8/2 treated MI group (MI+r*Eg*AgB8/2) compared to the untreated group after MI surgery (**Figure 2C**). By comparing the infarct area of mouse heart staining with TTC, the r*Eg*AgB8/2 treated MI group had significantly less infarct area than those without treatment (**Figure 2D**). The echocardiogram in long-axis B-mode revealed that untreated MI mice (MI+PBS) exhibited significantly reduced anterior and posterior wall motion of the left ventricle compared with MI mice treated with r*Eg*AgB8/2 (MI+r*Eg*AgB8/2). Treatment with r*Eg*AgB8/2 also significantly improved LVEF, LVFS, and SV in MI mice than those without treatment even though both groups maintained the similar heart rate (400–500 bpm) without significant difference (**Figure 2E**).

### 3.3 Treatment with r*Eg*AgB8/2 alleviated myocardial fibrosis and promoted tissue repair in MI mice

**Figure 3:**
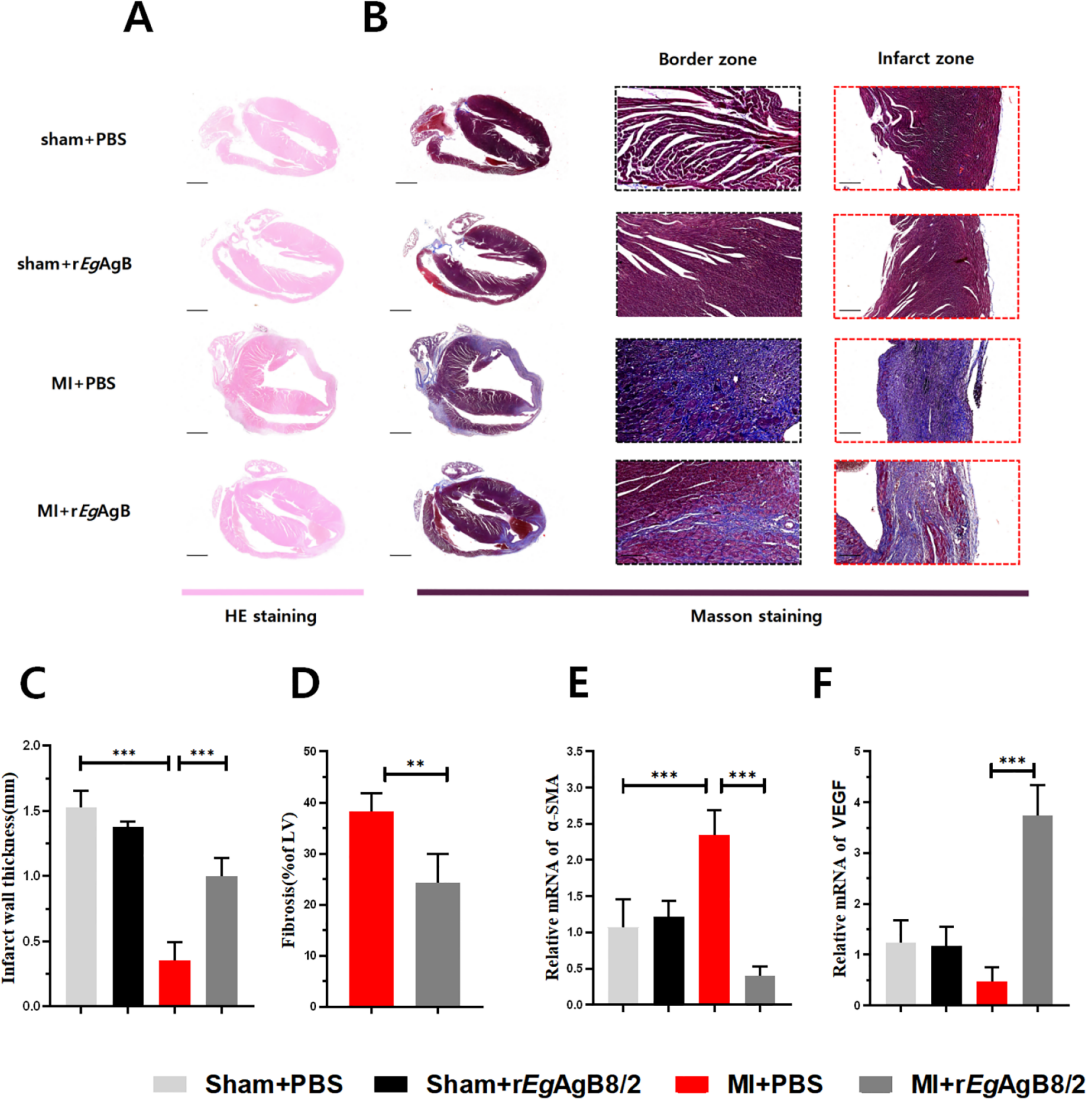
Treatment with r*Eg*AgB8/2 alleviated myocardial fibrosis and promoted tissue repair in MI mice. **(A)** Representative images of longitudinal sections of mouse hearts stained with H&E for each group. (Scale bar=1000μm). **(B)** Representative sections of Masson staining of heart tissue (Scale bar=1000μm) and enlarged views of infarct zone (Scale bar=250μm) in each group after MI. **(C)** Infarct wall thickness of the left ventricle in each group (n=5). **(D)** Fibrosis area of the infarction zone (IZ) in the MI group (n=5). **(E)** mRNA expression levels of α-SMA in the infarction zone (n=5). **(F)** mRNA expression levels of VEGF in the infarct region of the heart in each treated group (n=5). The results are presented as mean ± SEM, *P< 0.05, **P< 0.01, ***P< 0.001.

H&E (**Figure 3A**) and Masson’s trichrome staining (**Figure 3B**) on affected heart tissue demonstrated that r*Eg*AgB8/2 treatment significantly increased the thickness of the left ventricular wall (**Figure 3C**) and reduced myocardial fibrosis in mice with MI (**Figure 3D** **).** To determine if r*Eg*AgB8/2 is able to mitigate myocardial fibrosis and facilitate vascular reconstruction post-MI, α-SMA, a key marker for the activation of fibroblasts into myofibroblasts (Gabbiani, 2021), and VEGF, which is critical for vascular reconstruction and promotes repair of the infarcted myocardium (Laakkonen et al., 2019), were measured in the heart tissue. RT-qPCR results indicated the mRNA expression of α-SMA in the infarcted heart tissue was significantly decreased (**Figure 3E**) and VEGF transcription was increased (**Figure 3F**) in the cardiac tissues of MI mice treated with r*Eg*AgB8/2 compared to the mouse group without treatment (PBS). Together, these findings suggest that treatment with r*Eg*AgB8/2 significantly reduced myocardial fibrosis and improved heart tissue repair in mice following MI.

### 3.4 Treatment with r*Eg*AgB8/2 increased the expression of genes related to M2 macrophage and heart tissue repair detected by single-cell sequencing

**Figure 4:**
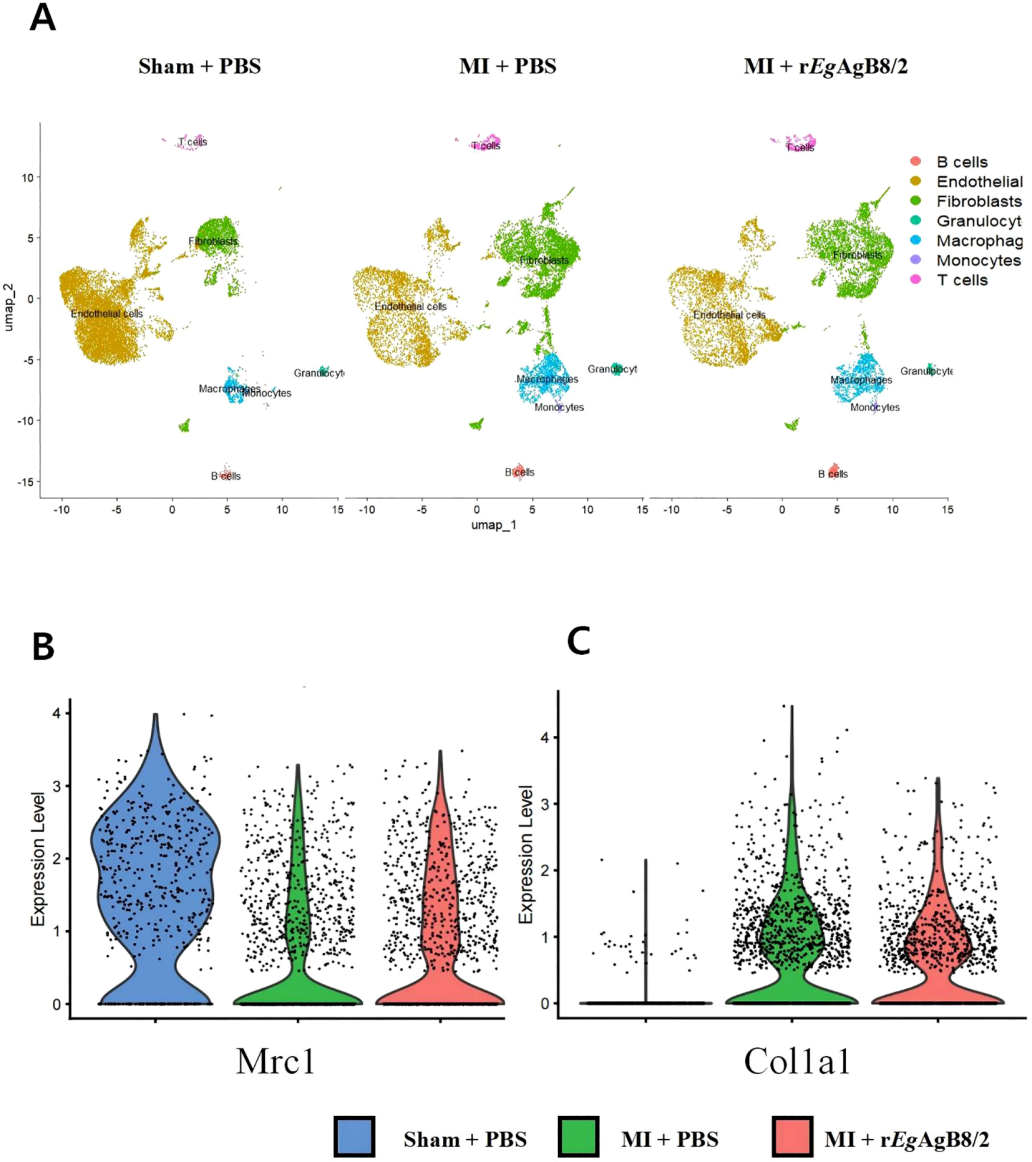
Treatment with r*Eg*AgB8/2 can regulate macrophage polarization and fibrosis levels in the mouse heart. **(A)** The UMAP plot generated using single-cell RNA sequencing technology illustrates the distribution of different cell types in heart tissues. **(B)** Violin plot of *Mrc1* gene variation in the hearts of mice from different groups. **(C)** Violin plot of *Col1a1* gene variation in the hearts of mice from different groups.

The uniform manifold approximation and projection (UMAP) plot generated using single-cell RNA sequencing technology shows the distribution of different cell types in the hearts of mice. The results indicate a significant increase in the number of fibroblasts and macrophages in MI mice. The rise in fibroblasts reflects the heart’s attempt to repair the damage, while the increase in macrophages is closely associated with the inflammatory response. After being treated with r*Eg*AgB8/2, the number of fibroblasts slightly decreased, whereas the number of macrophages remained at a high level (**Figure 4A**). However, the expression of *Mrc1* gene, a marker for M2 macrophages (Gordon and Martinez, 2010), was significantly increased compared to MI group without treatment, suggesting r*Eg*AgB8/2 induces the transition of macrophages to the M2 phenotype, thereby exerting anti-inflammatory and reparative effects in the heart with MI (**Figure 4B**). The expression of *Col1a1*, which encodes collagen type I (Myllyharju and Kivirikko, 2004), was also decreased, indicating that r*Eg*AgB8/2 inhibits excessive collagen synthesis, thereby reducing the risk of cardiac fibrosis (**Figure 4C**).

### 3.5 Treatment with r*Eg*AgB8/2 reduced inflammatory responses in infarcted heart tissue by inducing M2 macrophage polarization

**Figure 5:**
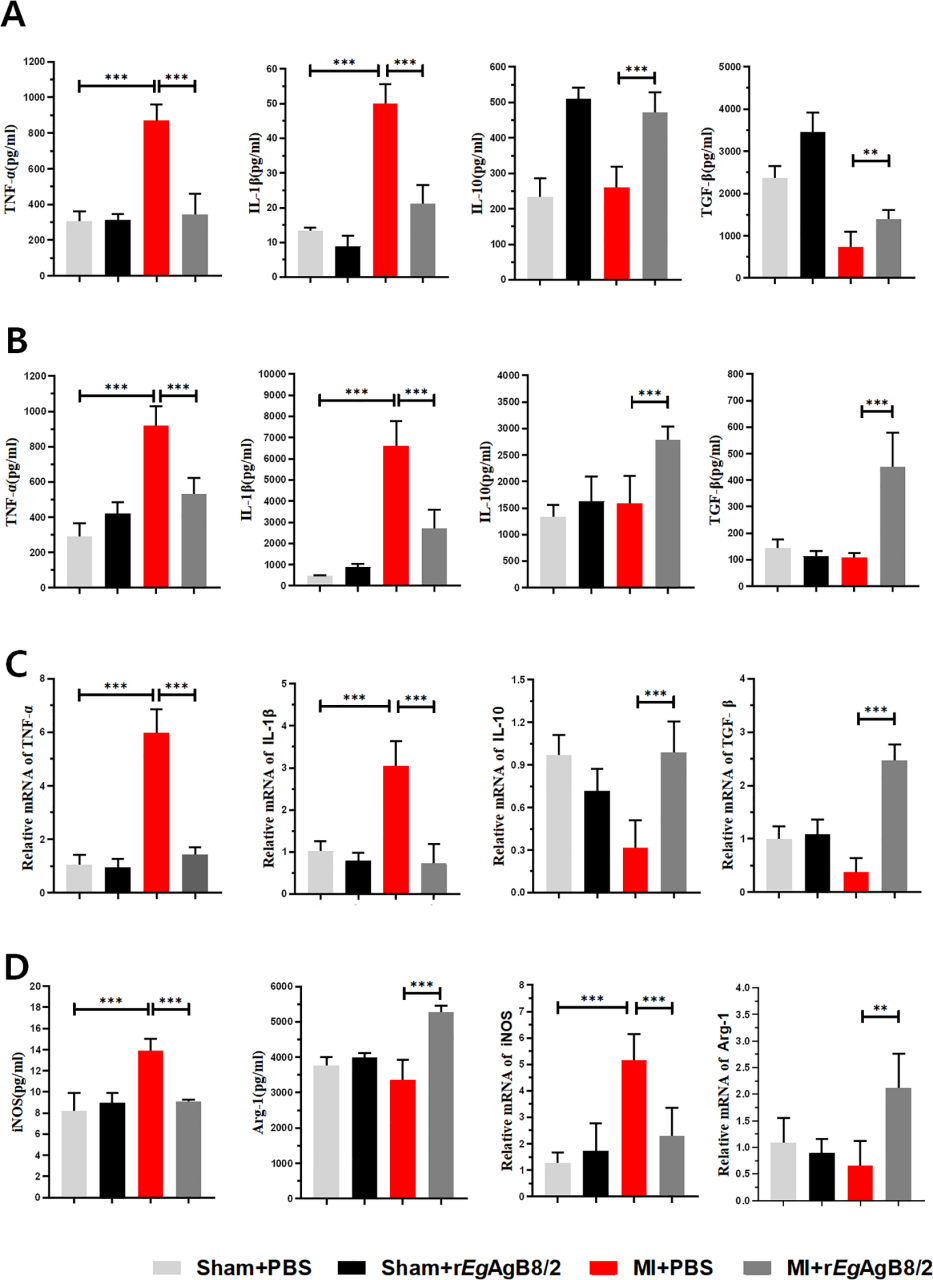
Treatment with r*Eg*AgB8/2 reduced the pro-inflammatory cytokine responses and promoted the polarization of macrophages to the M2 type in MI mice. **(A)** Treatment with r*Eg*AgB8/2 reduced the pro-inflammatory cytokine levels (TNF-α and IL-1β) and increased IL-10 and TGF-β levels in sera of mice with MI (TNF-α n=6; IL-1β n=5; IL-10 n=6; TGF-β n=6). **(B)** Treatment with r*Eg*AgB8/2 reduced the pro-inflammatory cytokine levels (TNF-α and IL-1β) and increased IL-10 and TGF-β in the infarct region of the mice with MI (n=5). **(C)** Treatment with r*Eg*AgB8/2 reduced the mRNA levels of inflammatory factors TNF-α and IL-1β and increased IL-10 and TGF-β in the infarct tissue of mice with MI (n=5). **(D)** Treatment with r*Eg*AgB8/2 decreased the expression in iNOS (M1 macrophage marker) and increased the expression of *Arg-1* (M2 macrophage marker) in MI mice (n=5). The results are presented as mean ± SEM, *P< 0.05, **P< 0.01, ***P< 0.001.

To determine whether the improvement in tissue damage in MI mice by r*Eg*AgB8/2 is associated with the modulation of cardiac inflammatory cytokines, we measured the levels of pro-inflammatory cytokines TNF-α and IL-1β, as well as regulatory cytokines IL-10 and TGF-β in mouse serum (**Figure 5A**) and in the MI tissue extracts (**Figure 5B**) using corresponding ELISA kits. The results showed that after treatment with r*Eg*AgB8/2, the levels of pro-inflammatory factors TNF-α and IL-1β was significantly decreased, while the levels of IL-10 and TGF-β significantly increased in both sera and MI tissues of the mice with MI. We also measured the mRNA levels of these pro-inflammatory cytokines and regulatory cytokines in MI tissues and the results were consistent with the protein levels measured by ELISA (**Figure 5C** **).** To determine the changes in macrophage phenotypes after r*Eg*AgB8/2 treatment, we detected both protein and mRNA expression levels of M1 macrophage marker iNOS and the M2 macrophage marker *Arg-1* in the infarcted heart tissues. The results revealed that treatment with r*Eg*AgB8/2 significantly decreased both protein and mRNA levels of iNOS and increased both protein and mRNA levels of *Arg-1* in the MI tissue (**Figure 5D**), indicating the reduced heart tissue inflammation and damage is possibly related to the polarization of macrophage from M1 type to M2 type in affected heart tissue.

### 3.6 Treatment with r*Eg*AgB8/2 inhibited the expression of NLRP3/caspase-1/IL-1β in the heart tissues of MI mice

**Figure 6:**
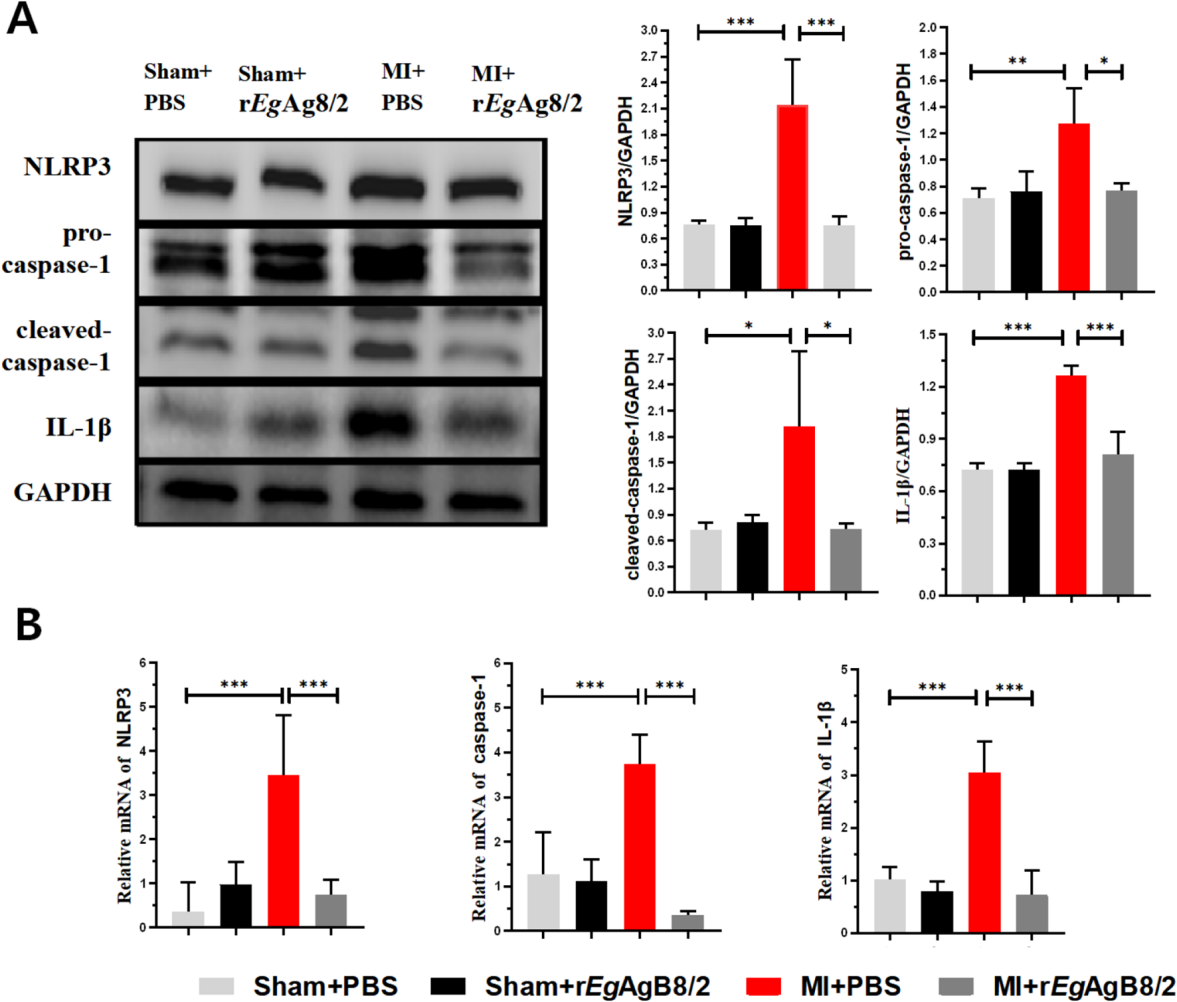
Treatment with r*Eg*AgB8/2 inhibited the NLRP3/caspase-1/IL-1β signaling pathway in the heart tissues of MI mice. GAPDH was used as a control. **(A)** Western blot results show that r*Eg*AgB8/2 reduced the expression levels of NLRP3, caspase-1, and IL-1β proteins in heart tissues of MI mice. The expression levels relative to GAPDH control was shown on the right (n=3). **(B)** Treatment with r*Eg*AgB8/2 reduced the mRNA levels of NLRP3, caspase-1, and IL-1β in the infarct region of mice with MI (n=5). The results are presented as mean ± SEM, *P< 0.05, **P< 0.01, ***P< 0.001.

Since NLRP3 (NOD-like receptor family pyrin domain-containing 3) inflammasome is an important component of the innate immune system that stimulates caspase-1 activation and the secretion of proinflammatory cytokines IL-1β/IL-18 in response to inflammation and cellular damage (Kelley et al., 2019), we measured if NLRP3/caspase-1/IL-1β signal pathway is involved in the therapeutic effect of r*Eg*AgB8/2 in MI mice. Western blot with specific antibodies demonstrated the protein expression of NLRP3, caspase-1, and IL-1β in the cardiac tissue of MI mice were significantly increased compared to the sham-operated group, indicating that NLRP3, caspase-1, and IL-1β were involved in the inflammatory response of MI. After treatment with r*Eg*AgB8/2, the expression levels of NLRP3, caspase-1, and IL-1β were significantly reduced (**Figure 6A**). We also utilized RT-qPCR to measure the transcriptional levels of the NLRP3, caspase-1, and IL-1β genes in the affected cardiac tissues of mice from each group, the RT-qPCR results were consistent with those of the Western blot analysis (**Figure 6B**). These results suggest that the inhibition of the NLRP3/caspase-1/IL-1β signaling pathway plays a key role in the therapeutic process of r*Eg*AgB8/2 in MI mice.

### 3.7 Treatment with r*Eg*AgB8/2 induced M2 macrophage polarization *in vitro*

**Figure 7:**
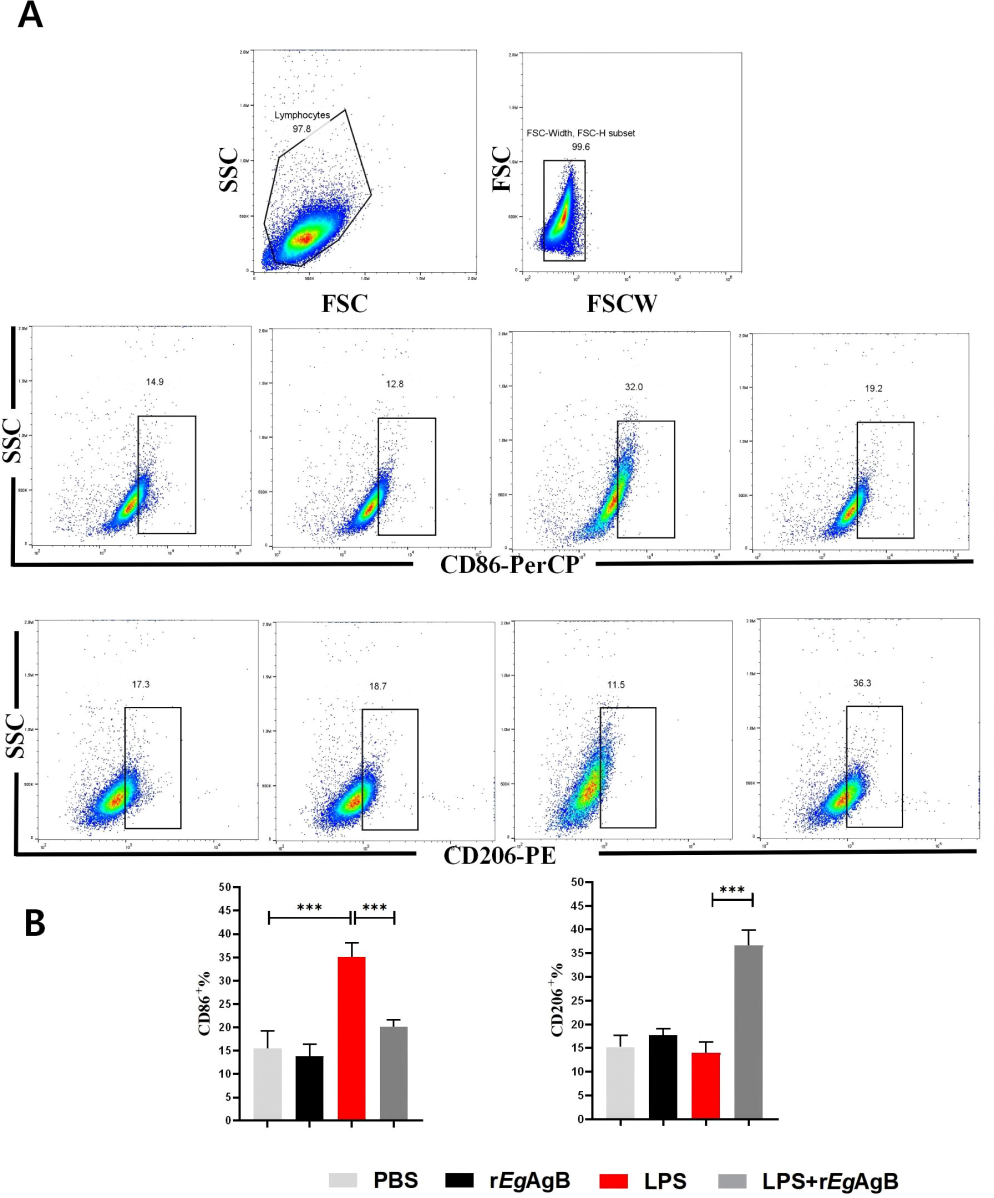
Co-incubation with r*Eg*AgB8/2 inhibited LPS-induced differentiation of RAW264.7 cells into M1 macrophages and promotes their differentiation into M2 macrophages. **(A)** A flow cytometry gating strategy was used to differentiate adherent cells. **(B)** Flow cytometer was used to measure the levels of M1 macrophage marker CD86 and M2 macrophage marker CD206 (n=3). The results are presented as mean ± SEM, *P< 0.05, **P< 0.01, ***P< 0.001.

**Figure 8:**
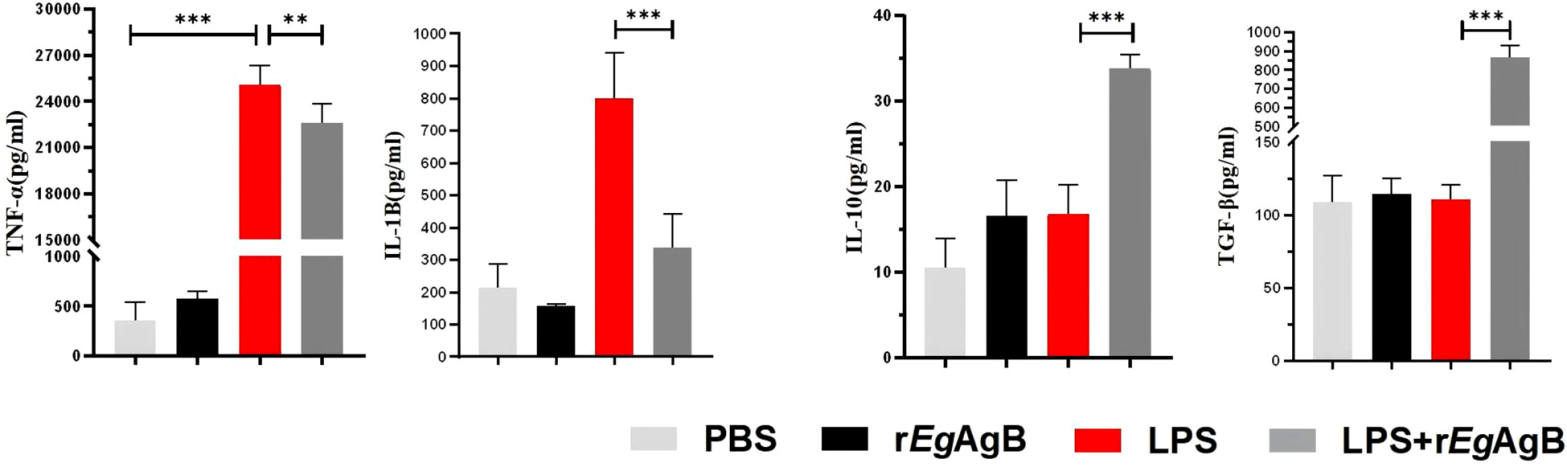
The M1 and M2 cytokine profile secreted by the RAW264.7 cells co-incubated with r*Eg*AgB8/2. ELISA method was used to detect the levels of M1 cytokines TNF-α and IL-1β, and M2 cytokines IL-10 and TGF-β in the supernatants of RAW264.7 cells from different incubation groups (n =5). The results are presented as mean ± SEM, *P< 0.05, **P< 0.01, ***P< 0.001.

To further determine the effects of r*Eg*AgB8/2 on the differentiation of macrophage, the macrophage cell line RAW264.7 was co-cultured with r*Eg*AgB8/2 *in vitro* to mimic the inflammatory microenvironment of macrophage in infarct heart tissue (Yan et al., 2013; Frangogiannis, 2014; Prabhu and Frangogiannis, 2016). LPS was added into the cultured RAW264.7 cells to induce the inflammatory responses when r*Eg*AgB8/2 was added (Zhang et al., 2023; Wang et al., 2024). Flow cytometry was used to measure the expression of the M1-related inflammatory marker CD86 and the M2-related marker CD206 in RAW264.7 cells after being co-incubated with r*Eg*AgB8/2. The results showed that LPS significantly induced expression of CD86+ in RAW264.7 cells. Co-incubation with r*Eg*AgB8/2 reduced the macrophages expressed with CD86+ and induced cells expressed with CD206+ (**Figures 7A, B**) compared to the group without r*Eg*ArB8/2 treatment, indicating r*Eg*AgB8/2 induces the macrophage polarization from M1 to M2 subtype. ELISA was used to measure the levels of inflammatory cytokines in the culture supernatants of each group after co-incubation. Compared to the PBS group, LPS stimulation increased the secretion of pro-inflammatory cytokines TNF-α and IL-1β in RAW264.7 cells. Co-incubation with r*Eg*AgB8/2 reduced the levels of the pro-inflammatory cytokines and increased the levels of the anti-inflammatory cytokines IL-10 and TGF-β (**Figure 8** **),** further confirming r*Eg*AgB8/2 promotes LPS-induced M1 macrophages transformation towards M2 *in vitro*.

## 4 Discussion

MI is the leading cause of death among cardiovascular diseases and has a significant impact on global public health (Salari et al., 2023). Studies have shown that the inflammatory response following MI is a major cause of further damage to heart tissue. Macrophages, as a powerful group of immune cells, play an important role in inflammation after MI. In the initial stages of MI, pro-inflammatory M1 macrophages predominate, leading to oxidative stress and tissue damage, exacerbating the inflammatory process. In the later stages of a heart attack, anti-inflammatory M2 macrophages replace M1 macrophages, promoting angiogenesis, alleviating tissue hypoxia, reducing myocardial injury, and accelerating ventricular repair (Zhang et al., 2022). Therefore, regulating the transition of macrophage to M2 subtype is crucial to the development and progression of post-infarction inflammation and the prognosis of MI.

To understand the dynamics of pathological progress and its relation with immune responses of MI, we successfully established a mouse model of MI by ligating the LAD coronary artery in mice as described (Gao et al., 2010). After ligating the LAD artery, mice became very sick and functions of left ventricular contraction became significantly weaken revealed by the reduced LVEF and LVFS values (**Figure 2**) and high death rate. All results indicate that we have successfully established the MI model in mice. Pathological examination displayed the edema of infarcted heart (increased heart/body weight ratio), thinner ventricular wall and increased myocardial fibrosis (**Figure 3**). At the early stage of MI, neutrophils and monocytes migrated to the infarcted area, driving the onset of the inflammatory response with high expression of proinflammatory cytokines (TNF-α and IL-1β) at both mRNA and protein level (**Figures 5B, C**). At the meantime, the M1 macrophage expressed iNOS was also significantly increased (**Figure 5D**). Seven days post-MI, the results of single-cell sequencing indicate that the clusters of macrophages and fibroblasts in the infarcted area of the hearts of MI mice were significantly increased compared to the sham-operated group, indicating the M1 macrophages are involved in the immune responses at the early stage of MI (Lv et al., 2017; Li et al., 2021) (**Figures 4A**, **5**). Excessive inflammation can lead to the degradation of the extracellular matrix, death of myocardial cells, impaired cardiac contractile function, and severe consequences such as cardiac rupture and failure (Nah and Rhee, 2009). Therefore, regulating the balance between M1 and M2 macrophage phenotypes and promoting M1 to M2 polarization are the key to mitigate the inflammatory response following MI and repair the damaged heart tissue at the later stage.

Recent studies have shown that helminths and their derived proteins have therapeutic effects on a variety of inflammatory diseases, such as arthritis, colitis, sepsis, asthma, and Type I diabetes (Brum et al., 2014; Peón and Terrazas, 2016; Quinn et al., 2019; Gao et al., 2021; Jafari et al., 2021). *Eg*AgB is an immunodominant component secreted by hydatid cysts of *E. granulosus* (Folle et al., 2024). Previous studies revealed that *Eg*AgB affected a variety of innate immune cells including neutrophils, monocytes, and dendritic cells, and induced immature dendritic cells to polarize towards a Th2-type response, thereby regulating local inflammatory responses (Riganò et al., 2007). Recent studies suggested that *Eg*AgB may enter mammalian cells by endocytic pathways and bind to THP-1 macrophages through lipoprotein LDL and HDL receptors, inducing an anti-inflammatory phenotype (Silva-Álvarez et al., 2016; da Silva et al., 2018). It has been reported that *Eg*AgB was able to treat inflammatory bowel disease by regulating the differentiation of macrophages towards the M2 type (Boutennoune et al., 2012). Our previous studies have also confirmed that the cyst fluid of *E. granulosus* enabled to treat mouse sepsis and subsequent multiple organ damage by regulating macrophage polarization (Wang et al., 2023). In this study, we investigated the potential immunomodulatory effect of r*Eg*AgB8/2 (one of the 5 *Eg*AgB subunits with the strongest immunogenicity during infection (Ahn et al., 2015) on MI-induced inflammation and recovery in a mouse model.

**Figure 9:**
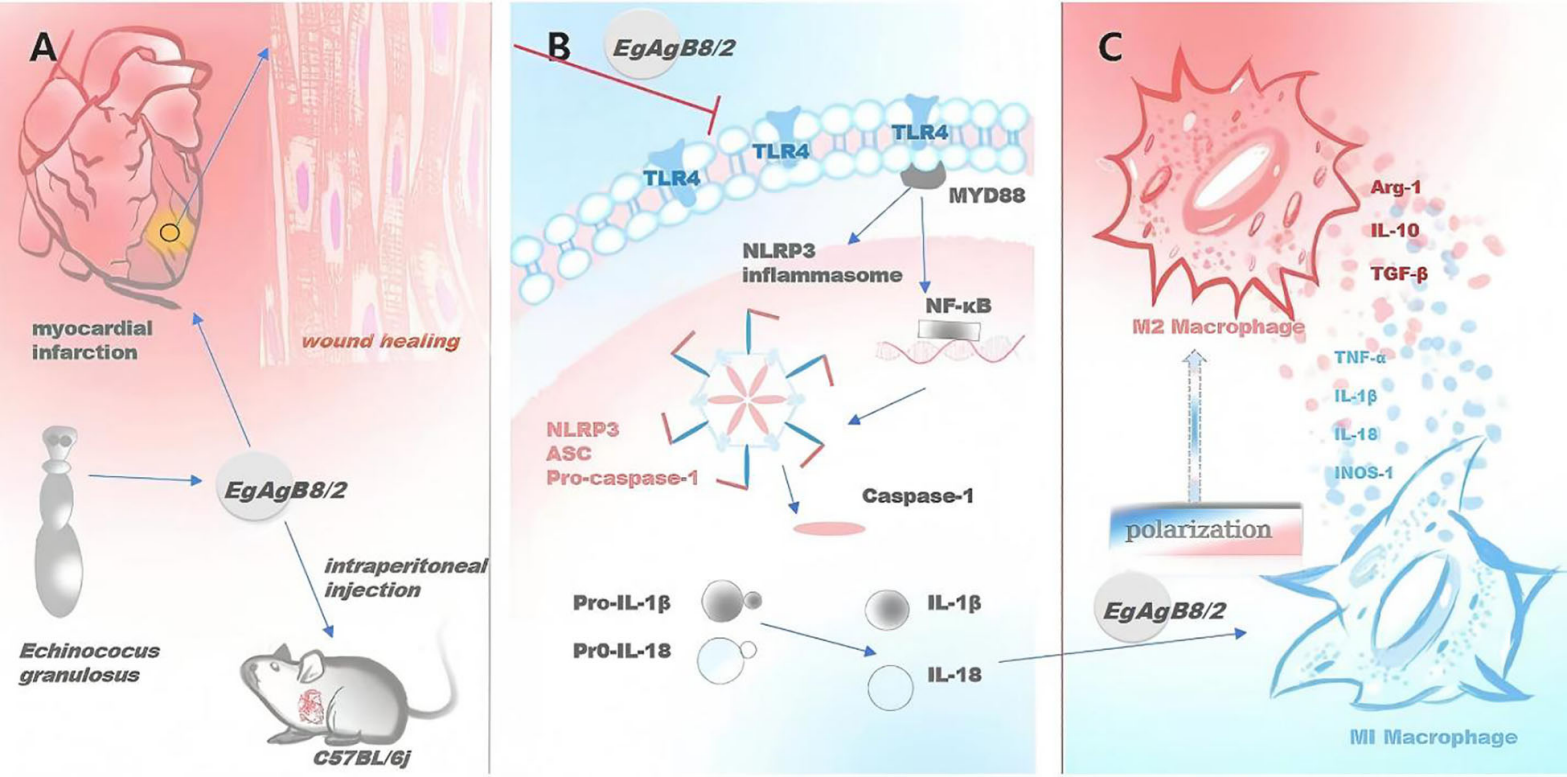
r*Eg*AgB8/2 alleviates MI and promotes tissue repair in MI mice through M2 macrophage polarization and NLRP3 inflammasome pathway. **(A)** r*Eg*AgB8/2 alleviates myocardial inflammation and promotes tissue repair in MI mice. **(B)** r*Eg*AgB8/2 inhibits the expression of proteins related to the inflammasome signaling pathway and regulates downstream inflammatory cytokines. **(C)** r*Eg*AgB8/2 induces the polarization of macrophages from M1 type to M2 type.

Our results demonstrated that r*Eg*AgB8/2 has a therapeutic effect on ischemia-caused MI in mice. Key manifestations of this therapeutic effect include enhanced cardiac function, increased survival rate, alleviated myocardial pathology, reduced tissue damage and fibrosis. The immunological and biochemical examination identified the improved MI was correlated with reduced inflammatory responses including reduced pro-inflammatory cytokines TNF-α and IL-1β and increased regulatory cytokines IL-10 and TGF-β. Further investigation revealed that reduction in MI caused sterile inflammation after r*Eg*AgB8/2 treatment was associated with macrophage polarization from pro-inflammatory M1 to regulatory M2 subtype and inhibition of NLRP3 inflammasome (**Figure 9**). These results were further confirmed by the co-incubation of r*Eg*AgB8/2 with LPS-activated macrophage RAW264.7 *in vitro* (**Figures 7**, **8**).

NLRP3 is the major component of inflammasome involved in the activation signal pathway triggered by infection and sterile injury (Martinon et al., 2002; Acevedo et al., 2023; Xu and Núñez, 2023). The effect of NLRP3 inflammasome on inflammation in MI has received increasing attention in recent years (Swanson et al., 2019; Zhang et al., 2022). Following MI, damaged cardiomyocytes release a series of damage-associated molecular patterns (DAMPs), which activate Toll-like receptors (TLRs) and initiate NLRP3 inflammasome expression (Toldo and Abbate, 2018). Subsequently, through mechanisms such as ionic imbalance and mitochondrial dysfunction, the NLRP3 inflammasome is further activated (Zhou et al., 2011). Under the combined influence of these conditions, the NLRP3 inflammasome complex is assembled with ASC (apoptosis-associated speck-like protein containing a CARD) and caspase-1. The activated caspase-1 cleaves pro-IL-1β and pro-IL-18 into their mature IL-1β and IL-18 (Toldo and Abbate, 2018; Chen and Xu, 2022). IL-1β and IL-18 then further activate immune cells, stimulate cytokine production and cause extracellular matrix conversion that exacerbate inflammation and tissue damage after MI (Kaplanski, 2018; Abbate, 2020). NLRP3 inflammasome also promotes pyroptosis (Li et al., 2020; Zhu et al., 2021) and oxidative stress (Nagoor Meeran et al., 2020) to dampen fibrosis (Qiu et al., 2018; Jiang et al., 2021) and devastate cardiac remodeling (Louwe et al., 2020). These processes collectively worsen cardiac function and accelerate heart failure progression following infarction. NLRP3 inflammasome is also involved in macrophage and related inflammatory responses. Studies have shown that NLRP3 inflammasome-related genes are upregulated in M1 macrophages derived from monocytes (Awad et al., 2017). Our results showed that NLRP3, caspase-1, and IL-1β expression was significantly upregulated in infarcted heart tissue compared to the sham group (**Figure 6**), confirming NLRP3 inflammasome activation post-MI. Several studies indicate that inhibiting activation of NLRP3 inflammasome promoted the polarization of macrophages from the pro-inflammatory M1 phenotype to the anti-inflammatory M2 phenotype, thereby mitigating many inflammatory diseases such as inflammatory root resorption (Zhang et al., 2020) and inflammation following peripheral nerve injury (PNI) (Sun et al., 2024). Notably, r*Eg*AgB8/2 treatment markedly reduced the expression of these three molecules in infarcted tissues (**Figure 6**). These findings highlight the impact of r*Eg*AgB8/2 on the macrophage polarization and the inhibition of inflammasome expression as well, leading to the reduced inflammatory responses and the improved infarction pathology. The similar results were also observed in the treatment of collagen-induced arthritis (CIA) with *ES-62*, a glycoprotein secreted by *filarial* nematodes that regulated oxidative stress through NRF2-mediated counter-regulation of the inflammasome and inhibited pro-inflammatory cytokine responses (Rzepecka et al., 2015). A protein secreted by a sheep nematode *Haemonchus contortus* interacted with the NLRP3 inflammasome activation-associated G protein and inhibited the maturation of IL-1β and IL-18 (Wu et al., 2023).

The strong evidences shown in this study suggest r*Eg*AgB8/2 is a strong immunomodulatory reagent that could be used for the treatment of hyper-inflammatory diseases such as MI caused heart inflammation and damage. Compared with existing clinical therapies for MI, r*Eg*AgB8/2 not only avoids tissue damage associated with reperfusion therapy but also overcomes the infection risks posed by the broad-spectrum immunosuppression of anti-inflammatory drugs. This dual synergistic effect of anti-inflammatory and pro-repair activities suggests the significant potential of r*Eg*AgB8/2 for clinical translation, offering novel therapeutic strategies for myocardial infarction and other inflammation-related diseases in future.

However, the exact mechanism underlying the immunomodulation of *Eg*AgB is not fully understood regarding the macrophage polarization and its relationship to the inflammasome inhibition. It is still unknown whether the *Eg*AgB8/2 stimulated M2 polarization is a result of NLRP3 inhibition or M2 polarization leads to the inhibition of inflammasomes. It needs to further investigate by creating a NLRP3 knockout model to understand the exact role of NLRP3 in immune response and macrophage regulation.

Except for NLRP3, it needs to be further investigated whether any other inflammasome or pathway is involved in macrophage polarization, such as NLRC4 or AIM2 inflammasomes, or upstream signaling molecules like *NF-κB*. The current studies for the immune therapy for inflammatory or autoimmune diseases with helminth infections or helminth-derived products, including r*Eg*AgB8/2 for reducing MI inflammation in this study, were mostly performed in experimental animal models. There is still long way to go with the transitional procedures to clinical trials including the cGMP production of immunomodulatory products, regulatory guideline and safety issues. Recently, clinical trials with hookworm infections for the treatment of allergic rhinitis (Blount et al., 2009) and for Type 2 diabetes (Pierce et al., 2019) in human were under investigation.

## 5 Conclusion

r*Eg*AgB8/2 display a promising therapeutic effect on MI by targeting the NLRP3 inflammasome pathway and modulating macrophage polarization. These findings lay the groundwork for further exploration of r*Eg*AgB8/2 and other helminth-derived proteins in the treatment of cardiovascular diseases associated with excessive inflammation and other inflammatory diseases.

## Data Availability

The datasets presented in this study can be found in online repositories. The names of the repository/repositories and accession number(s) can be found in the article/supplementary material.
